# Symptoms and laboratory manifestations of mild COVID-19 in a repatriated cruise ship cohort

**DOI:** 10.1017/S0950268821000315

**Published:** 2021-02-10

**Authors:** C. R. Bailie, L. Franklin, S. Nicholson, F. Mordant, C. Alpren, T. Stewart, C. Barnes, A. Fox, J. Druce, K. Subbarao, M. Catton, A. van Diemen, S. G. Sullivan

**Affiliations:** 1 Victorian Department of Health and Human Services, Communicable Diseases Epidemiology and Surveillance, Melbourne, VIC, Australia; 2 WHO Collaborating Centre for Reference and Research on Influenza, Royal Melbourne Hospital, At the Peter Doherty Institute for Infection and Immunity, Melbourne, VIC, Australia; 3 National Centre for Epidemiology and Public Health, Australian National University, Canberra, ACT, Australia; 4 Victorian Infectious Diseases Reference Laboratory, Royal Melbourne Hospital, At the Peter Doherty Institute for Infection and Immunity, Melbourne, VIC, Australia; 5 Department of Microbiology and Immunology, University of Melbourne, At the Peter Doherty Institute for Infection and Immunity, Melbourne, VIC, Australia; 6 National Incident Room, Commonwealth Department of Health, Canberra, ACT, Australia; 7 Doherty Department, University of Melbourne, At the Peter Doherty Institute for Infection and Immunity, Melbourne, VIC, Australia; 8 Health Protection Branch, Victorian Department of Health and Human Services, Melbourne, VIC, Australia

**Keywords:** Asymptomatic, COVID-19, cruise ship, SARS-CoV-2, serology

## Abstract

Much of our current understanding about novel coronavirus disease 2019 (COVID-19) comes from hospitalised patients. However, the spectrum of mild and subclinical disease has implications for population-level screening and control. Forty-nine participants were recruited from a group of 99 adults repatriated from a cruise ship with a high incidence of COVID-19. Respiratory and rectal swabs were tested by polymerase chain reaction (PCR) for severe acute respiratory syndrome coronavirus 2 (SARS-CoV-2). Sera were tested for anti-SARS-CoV-2 antibodies by enzyme-linked immunosorbent assay (ELISA) and microneutralisation assay. Symptoms, viral shedding and antibody response were examined. Forty-five participants (92%) were considered cases based on either positive PCR or positive ELISA for immunoglobulin G. Forty-two percent of cases were asymptomatic. Only 15% of symptomatic cases reported fever. Serial respiratory and rectal swabs were positive for 10% and 5% of participants respectively about 3 weeks after median symptom onset. Cycle threshold values were high (range 31–45). Attempts to isolate live virus were unsuccessful. The presence of symptoms was not associated with demographics, comorbidities or antibody response. In closed settings, incidence of COVID-19 could be almost double that suggested by symptom-based screening. Serology may be useful in diagnosis of mild disease and in aiding public health investigations.

## Background

The spectrum of novel coronavirus disease 2019 (COVID-19) ranges from asymptomatic infection to death due to respiratory failure or other complications. However, most cases appear to experience mild illness [1], with estimated case-hospitalisation rate varying from 0% to 18% depending on age [2]. Fever and cough are common symptoms in hospitalised patients [3]. In mild cases, fever is less common, and gastrointestinal (GI) symptoms, and loss of taste or smell are reported frequently [4–6]. A minority of studies examining symptom profiles have investigated non-hospitalised cases [4].

Respiratory viral shedding peaks around the time of symptom onset, then decreases, reaching the limit of detection by polymerase chain reaction (PCR) on average about 2 weeks later [7, 8]. Longer duration of shedding is correlated with more severe illness [8, 9]. Prolonged faecal PCR positivity has been reported in mild or asymptomatic illness, raising the possibility of faecal–oral transmission from undetected carriers [10, 11]. However, most attempts to recover virus from faecal samples or rectal swabs have been unsuccessful, and the role of faecal–oral transmission remains unclear [10, 12].

Use of serologic assays for the detection of antibodies against severe acute respiratory syndrome coronavirus 2 (SARS-CoV-2) has been suggested as an aid to clinical diagnosis, for estimation of population wide attack rates, and for retrospective investigation of transmission chains [13, 14]. Immunoglobulin (Ig) G assays have reported sensitivity of 85–95% at >14 days since symptom onset and specificity of 92–99%, while IgA assays are less specific but have higher sensitivity earlier in the disease course [15, 16]. However, serum used for validation has mostly come from symptomatic patients [15–17], and it is unclear whether these results are generalisable to people without symptoms. Emerging evidence suggests that asymptomatic cases may have a less robust IgG response and that antibody titres decay rapidly [18, 19]. Older age and presence of comorbidities predispose towards severe disease [20], and the risk of symptomatic disease appears to increase with older age [21]. However, other determinants of developing symptomatic *vs.* asymptomatic illness are not well understood.

Because outbreaks of COVID-19 on cruise ships occur in closed settings with high rates of exposure, they provide opportunities to study a broader spectrum of illness than that which may be apparent from active or passive case finding in the community [22]. On 3 April, all 217 people on board a cruise ship off the coast of Uruguay known to have COVID-19 cases were tested for SARS-CoV-2. Fifty-nine percent tested positive [23]. Most were reported to be asymptomatic [23]. Passengers had been confined to their cabins from 22 March. On 12 April 2020, 99 adult passengers and crew were repatriated to Australia [23]. They were separated according to their test results on the repatriation flight. On arrival in Melbourne, Australia, all were required to undertake 14 days of isolation or quarantine in a designated hotel.

This study aimed to describe the attack rate, symptoms, viral shedding patterns and serologic response in this cohort of Australian returned travellers, to investigate possible determinants of symptomatic illness, and to examine differences in antibody response between symptomatic and asymptomatic cases.

## Methods

### Public health response

Because of the high proportion of passengers and crew reported to have tested positive in Uruguay, all returned travellers were treated as suspected cases upon arrival in Melbourne. They were interviewed by phone to collect demographic information, information on relevant symptoms and past medical history. They were also asked to provide copies of letters they had received stating their PCR test result from Uruguay. Victorian authorities subsequently did not accept these letters confirming infection status as proof of infection because laboratory reports were not included. Therefore, all returned travellers were requested by the public health authority to provide a nasopharyngeal swab for SARS-CoV-2 testing. These swabs were collected between days 1 and 7 after arrival in Australia. The state public health unit contacted returned travellers daily to monitor for signs and symptoms of COVID-19 until they were cleared from isolation or quarantine.

During their interviews, the returned travellers were invited to participate in the study. Participants provided consent for the study team to access data collected in routine case follow-up, including their PCR test results, and for the collection of additional biospecimens. The study was approved by the Human Research Ethics Committee of the Department of Health and Human Services, Victoria (HREC 05-20).

### Data collection

Data collected as part of case follow-up were abstracted from the Department of Health and Human Services' Public Health Events Surveillance System. Nurse-collected respiratory swabs (nasopharyngeal and pharyngeal) and self-collected rectal swabs were requested on recruitment, if not already provided. Participants with an initial PCR-positive respiratory swab were asked to provide follow-up swabs every 1–2 days until they returned two consecutive negative swabs, or reached the end of their isolation or quarantine period, whichever occurred sooner. Results of additional swabs collected for public health or clinical reasons during the isolation or quarantine period were collated and included in the analysis. Two blood samples were requested from each participant, the first on either 16 April or 17 April, and the second on 24 April.

### Virus characterisation

Respiratory and rectal swabs were tested for the presence of SARS-CoV-2 RNA using real-time PCR (RT-PCR) targeting the RdRp, E and N genes [24]. Virus isolation in Vero cells was attempted for all PCR-positive samples [24].

### Serology

Sera were tested using commercial kits manufactured by EUROIMMUN AG (Lübeck, Germany) for the detection of IgA and IgG by enzyme-linked immunosorbent assay (ELISA), using the S1 domain of the SARS-CoV-2 spike protein as antigen, according to the manufacturer's instructions [25]. For these kits, the ratio between the extinction value for the sample and calibrator provides a semi-quantitative measure of antibody response (14). The sensitivity of the IgA and IgG kits using sera collected between 10 and 21 days after symptom onset was calculated by the manufacturer as 100.0% and 87.5%, respectively, with the sensitivity of the IgG assay increasing to 100.0% for samples collected at least 21 days after symptom onset [25, 26]. Specificity was reported as 90.5% and 99.3%, respectively [25, 26].

An in-house microneutralisation (MN) assay was used to detect neutralising antibody against SARS-CoV-2. SARS-CoV-2 isolate CoV/Australia/VIC01/2020 passaged in Vero cells was stored at −80 °C. Serial twofold dilutions of heat-inactivated serum were incubated with 100 median tissue culture infectious doses (TCID_50_) of SARS-CoV-2 for 1 h and residual virus infectivity was assessed in quadruplicate wells of Vero cells; viral cytopathic effect was read on day 5. The neutralising antibody titre was calculated using the Reed/Muench method. Based on prior validation of this assay using SARS-CoV-2 positive and negative (pre-2019 sera), a titre of 40 or more was taken to indicate a positive antibody response. The sensitivity and specificity of this assay at this threshold were estimated at 70% (95% confidence interval (CI): 55–82%) and 74% (95% CI 60–85%), respectively (unpublished data).

### Statistical methods

Data analyses were performed in R version 3.6.1. Because timing of swab collection correlated poorly with symptom onset, cases were defined as participants with evidence of any positive PCR result, or a positive IgG result from either blood sample. The attack rate was calculated using the total number of participants as the denominator. Summary statistics for presence and timing of symptoms were calculated for symptomatic cases. Pearson correlation coefficients (*r*) were calculated to examine correlation between MN titres and ELISA results. Two-sided Mann–Whitney *U* tests were performed to test the hypotheses that the distributions of MN titres and ELISA results differed between symptomatic and asymptomatic PCR positive participants, at each time point.

For participants meeting the study case definition, the relationships between the presence of symptoms as outcome, and binary predictors (sex, age group and presence of comorbidities), were examined independently. After constructing binary dummy variables for each age group, the risk of symptoms was calculated for cases with and without each predictor. Relative risk was calculated as the ratio between these two values. The ‘EpiStats’ package was used to calculate two-sided 95% CIs, and to compute *P*-values using chi-squared tests.

## Results

**Fig. 1.**
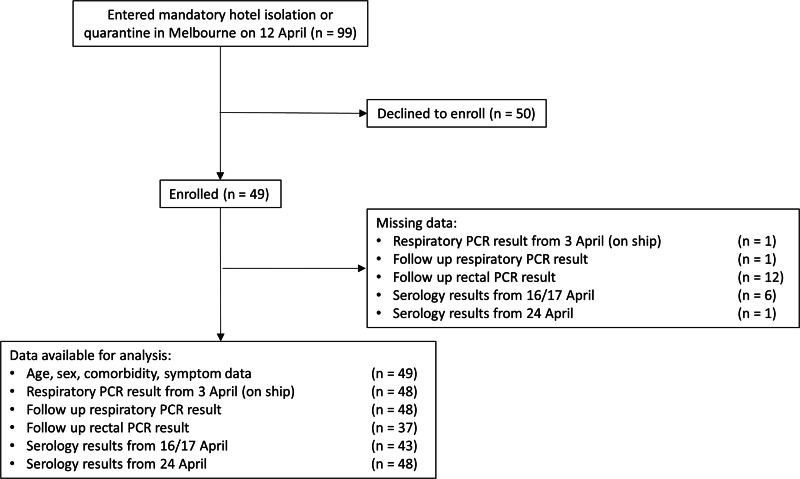
Flow diagram showing enrolment of cohort, data available for analysis and missing data.

Forty-nine participants were recruited from the 99 Australians repatriated to Melbourne (49%, Fig. 1). Forty-three were initially recruited, with a further six recruited for follow-up blood collection only. Demographic and health information are summarised in Table 1. The median age was 67 years (range: 36–81), and 31 (63%) were female. Nearly half of participants reported a comorbid condition. One participant was hospitalised in Uruguay. Twenty-seven participants (55%) reported symptoms either on the ship or in hotel quarantine.

**Table 1.** Demographics, comorbidities, hospitalisation, presence of symptoms and previous SARS-CoV-2 PCR results for study participants (*n* = 49)

Age in years – median (range)	67 (36–81)
Sex	
Female – *n* (% of total)	31 (63.3)
Male – *n* (% of total)	18 (36.7)
Comorbidities	
None – *n* (% of total)	26 (53.1)
Any – *n* (% of total)	23 (46.9)
Chronic respiratory disease – *n* (% of total)	6 (12.2)
Cardiac disease (excluding uncomplicated hypertension) – *n* (% of total)	5 (10.2)
Other – *n* (% of total)	19 (38.8)
Symptoms	
Present – *n* (% of total)	27 (55.1)
Absent – *n* (% of total)	22 (44.9)
Hospitalised	
Yes – *n* (% of total)	1 (2.0)
No – *n* (% of total)	48 (98.0)
Respiratory PCR result on ship (3 April 2020)	
Positive – *n* (% of total)	36 (73.5)
Negative – *n* (% of total)	12 (24.5)
Not available – *n* (% of total)	1 (2.0)

**Fig. 2.**
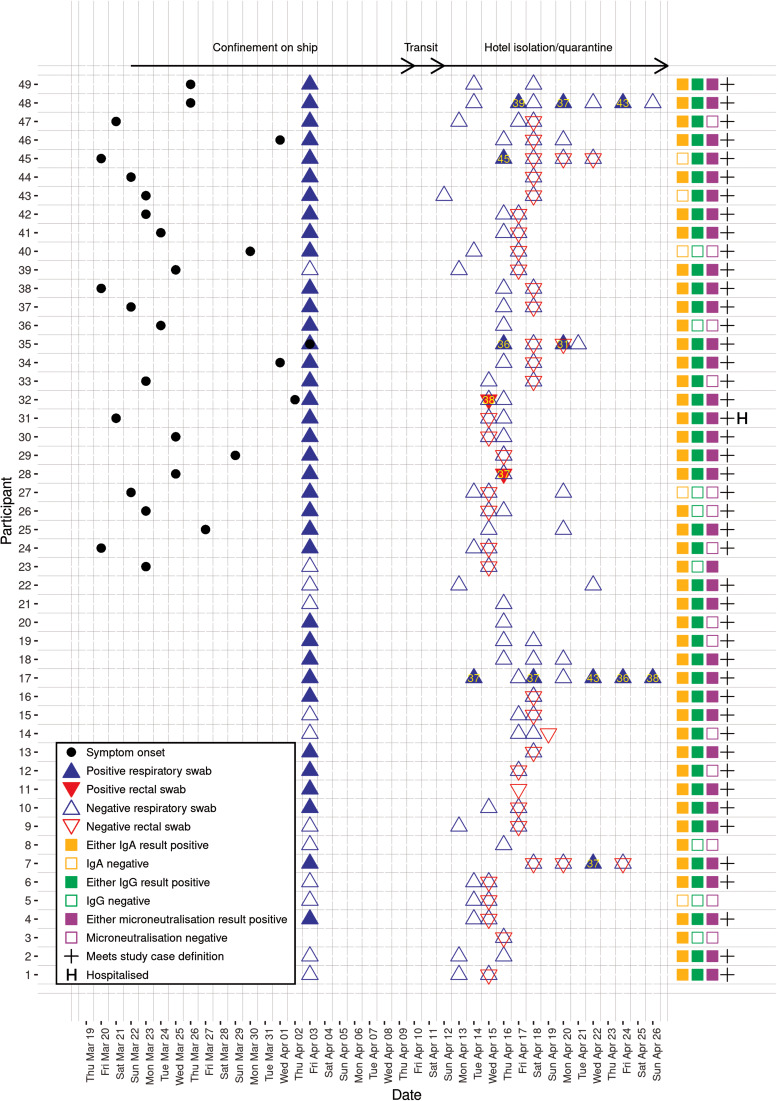
Timing of symptom onset, SARS-CoV-2 PCR testing and anti-SARS-CoV-2 serology, for 49 study participants. Participants were members of a cohort exposed to SARS-CoV-2 on board a cruise ship. Yellow numerals show cycle threshold (Ct) values. Serology results, case status and hospitalisation are presented on the right-hand side of the plot for readability, blood samples were collected on 16–17 April 2020 and 24 April 2020. Ig, immunoglobulin.

Testing reports from Uruguay were provided by 48 participants, of whom 36 were stated to have tested positive (73% of total). Forty-eight participants provided at least one respiratory swab for testing in Melbourne, with the first collected a median 23 days (range: 13–27 days) after symptom onset. Five (10%) tested positive (Fig. 2), but only three of these five reported symptoms on the ship or during the follow-up period. By the end of the 14-day quarantine period, only one of these participants had returned two consecutive negative swabs.

Thirty-seven participants provided rectal swabs, two of which were positive (5%). Respiratory swabs collected from these two participants on the same date were negative. Both had previously experienced respiratory symptoms, but neither reported diarrhoea. For the 14 PCR-positive respiratory and rectal swabs collected from the seven participants who were PCR positive in Melbourne, the median cycle threshold (Ct) value for the RdRp gene was 37 (range: 31–45). Virus isolation was unsuccessful for all samples.

**Fig. 3.**
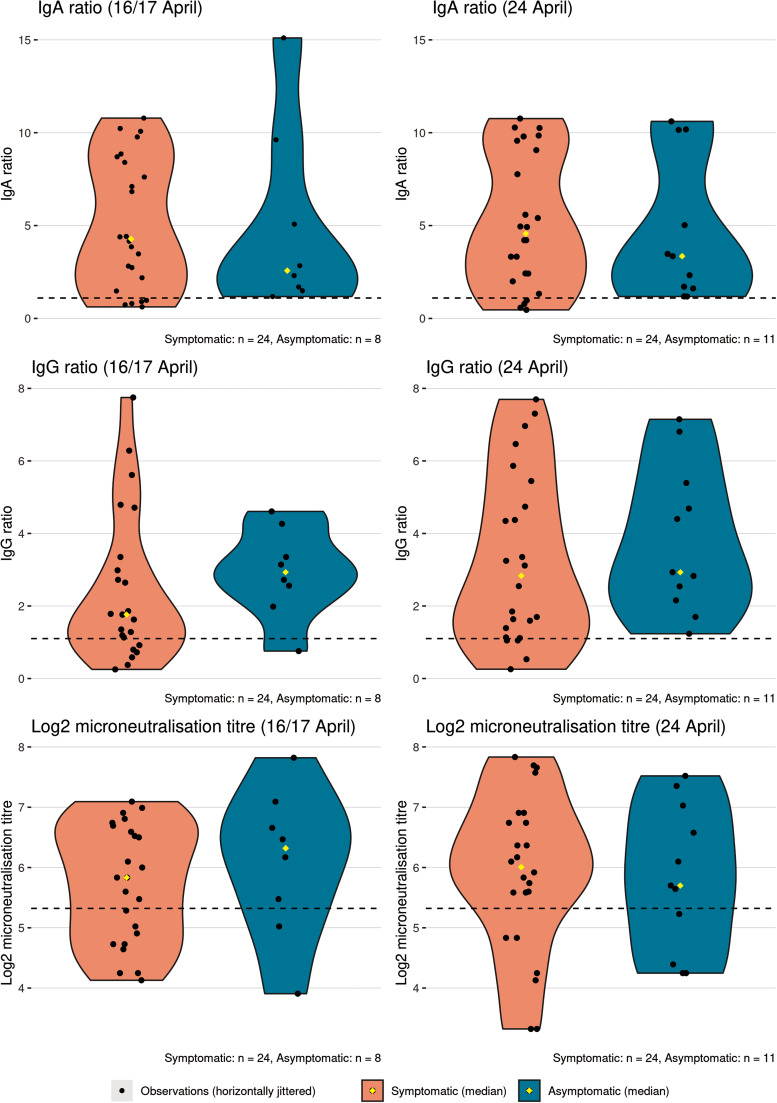
Distribution of SARS-CoV-2 serology results in 36 study participants testing positive by PCR, by the presence of symptoms. Participants were members of a cohort exposed to SARS-CoV-2 on board a cruise ship. Median symptom onset was 24 March 2020. Dashed lines show threshold above which a result is considered positive. Ig, immunoglobulin.

Consecutive blood samples were collected a median of 24 and 31 days after symptom onset, respectively. There was wide variation in antibody responses, even among PCR-negative participants (Fig. 3, Supplementary figure). For the 42 participants who provided two blood samples, the number with a positive IgA result increased from 36 (86%), on the first sample to 37 (88%) on the second sample, while the number with a positive IgG result increased from 30 (71%) to 34 (81%). MN assay positivity increased from 28 (67%) to 30 (71%). The MN was highly correlated with the IgG results (*r* = 0.85), and less well with the IgA (*r* = 0.6, data not shown).

Despite a trend towards higher IgA, and lower IgG response in symptomatic *vs.* asymptomatic PCR-positive participants, the overall distribution of IgA and IgG ratio and log_2_ MN titre appeared similar in both groups for samples collected 24 and 31 days after median symptom onset (Fig. 3). No statistically significant differences were detected between groups in the results of any assay on either sample (two-sided Mann–Whitney *U* test, IgA: *P* = 0.85, *P* = 0.76; IgG: *P* = 0.21, *P* = 0.38; MN: *P* = 0.49, *P* = 0.78; first and second samples, respectively).

Based on positive PCR or IgG results, 45 participants were estimated to have been infected, giving an attack rate of 92% among participants. However, 42% of cases reported no symptoms on the ship or during the follow-up period. One of the four study participants not meeting our composite case definition reported having been unwell with 1 day of fever in late March. The median date of symptom onset for the 26 symptomatic cases was 24 March (range: 20 March–3 April). Among the symptomatic cases, cough, sore throat, diarrhoea and headache were the most common symptoms (Table 2). Only four (15%) reported fever. Non-specific symptoms of lethargy or headache were the only symptoms reported by three cases (12%). Among the 45 cases, the presence of symptoms was not associated with age, sex or presence of comorbidities (Table 3).

**Table 2.** Symptom profiles in 26 symptomatic cases of mild COVID-19, disaggregated by sex

	Number reporting symptom (% of total)		
Symptom	All cases (*n* = 26)	Male cases (*n* = 8)	Female cases (*n* = 18)
Cough	15 (58%)	4 (50%)	11 (61%)
Sore throat	7 (27%)	2 (25%)	5 (28%)
Diarrhoea	6 (23%)	2 (25%)	4 (22%)
Headache^a^	6 (23%)	2 (25%)	4 (22%)
Runny/blocked nose^a^	5 (19%)	2 (25%)	3 (17%)
Lethargy^a^	5 (19%)	1 (12%)	4 (22%)
Fever	4 (15%)	1 (12%)	3 (17%)
Body aches^a^	4 (15%)	0 (0%)	4 (22%)
Loss of taste or smell^a^	3 (12%)	0 (0%)	3 (17%)
Shortness of breath	1 (4%)	0 (0%)	1 (6%)

a Participants were not routinely asked about these symptoms but volunteered them when asked about ‘other’ symptoms.

**Table 3.** Results of univariable analysis for cases of COVID-19 with presence of symptoms as outcome (*n* = 45)

Predictor	Number with predictor	Number with predictor symptomatic	Risk of symptoms with predictor (%)	Number without predictor	Number without predictor symptomatic	Risk of symptoms without predictor (%)	RR (95% CI)	*P* value (χ^2^)
Sex								
Female	29	18	62	16	8	50	1.24 (0.70–2.19)	0.43
Male	16	8	50	29	18	62	0.81 (0.46–1.42)	0.43
Comorbidities								
None	24	13	54	21	13	62	0.88 (0.53–1.44)	0.60
1 or more	21	13	62	24	13	54	1.14 (0.69–1.88)	0.60
Age group								
Age <55	6	5	83	39	21	54	1.55 (0.98–2.45)	0.17
Age 55–64	12	6	50	33	20	61	0.82 (0.44–1.55)	0.52
Age ≥65	27	15	56	18	11	61	0.91 (0.55–1.50)	0.71

RR, relative risk; CI, confidence interval; χ^2^, chi-squared test.

Of the 36 participants with a letter reporting a positive PCR result from Uruguay, 32 (89%) were IgG positive. Of the 12 with a letter reporting a negative result, three (25%) were IgG negative but nine (75%) were IgG positive, while the one participant who did not provide a letter was IgG negative.

## Discussion

This study synthesised symptoms, demographic and health data with the results of PCR and serology testing from a cohort of returned travellers exposed to SARS-CoV-2, among whom the attack rate was 92%. The asymptomatic fraction was 42%, similar to the estimated asymptomatic fraction on Diamond Princess [27], and of the remainder only one was hospitalised. Cough and sore throat were the most common symptoms. Self-reported fever was present in only 15% of symptomatic cases, underscoring the fallibility of temperature checks for screening [28].

A minority of participants had positive respiratory (10%) swabs when tested in Melbourne. This was not surprising as these tests were performed around 3 weeks after median symptom onset, and respiratory viral shedding has been reported elsewhere to persist for 1–2 weeks in most cases [7, 8]. That only 2 of 37 (5%) of participants had positive rectal swabs was somewhat unexpected given previous findings that about half of patients appear to shed RNA in faeces [10], for a median duration of almost 1 month after symptom onset [10]. This discrepancy might be due to the low disease severity in our cohort, or may be related to technique when self-collecting rectal swabs. Neither of the two participants with positive rectal swabs reported GI symptoms and both tested negative on respiratory swabs from the same day as rectal swab collection. In patients with positive rectal or stool samples, GI shedding tends to persist after clearance from the upper respiratory tract [10, 12] and only a minority report GI symptoms [10].

Although four participants continued to shed virus at the end of their hotel period, the high Ct values and inability to isolate live virus suggest the risk of onward transmission was low. The study team was notified by two of these cases that they tested positive again roughly 5 weeks after their first positive tests. Persistent viral shedding has been reported elsewhere [10]. In a Korean report of 285 cases who had a repeat positive PCR result after being released from isolation, there were no confirmed episodes of onward transmission after isolation, live virus isolation was unsuccessful in all 108 cases where it was attempted, and neutralising antibody was detected in the serum of all 23 cases who provided samples [29]. Persistent shedders present a problem for public health because it can be unclear whether they are at transmission risk and therefore require isolation. If isolation criteria are based solely on the timing of a positive PCR result, as may be the case for those without symptoms, it is possible that many people will be required to isolate unnecessarily.

At 1 month after median symptom onset, IgA and IgG ratios and MN titre were comparable between symptomatic and asymptomatic PCR-positive participants. This finding contrasts with recently published research, which found significantly lower IgG levels in 37 asymptomatic patients compared to 37 age- and sex-matched symptomatic patients during both the acute and early convalescent phases [19]. There are several possible explanations for this difference. First, because asymptomatic cases in that study were detected through testing of contacts of known cases, the interval from exposure to testing may have been shorter than for matched symptomatic cases, meaning asymptomatic cases would have had less time for a measurable IgG response to develop [19]. This is supported by the authors' finding that time from first to last positive PCR result was longer in the asymptomatic group. In our study, date of exposure should have been on average the same for symptomatic and asymptomatic participants. Second, asymptomatic patients in the abovementioned study were treated with α-interferon, ribavirin and additional supportive treatments according to local protocol [19], possibly clouding the natural history of disease.

This particular group of returned travellers presented a problem for public health authorities. Cruise ship outbreaks were particularly prominent in the media at the time after more than a hundred cases were identified linked to passengers allowed to disembark from Ruby Princess [30]. Participants in our study were not provided formal laboratory results confirming infection but few remained positive in Melbourne. IgG testing confirmed the overseas test reports for 35 participants and suggested a further nine had been infected despite the absence of a positive PCR report. In this cohort, there appears to have been utility in using serology to confirm infection, where the prevalence of disease was high and sufficient time had passed to permit development of detectable antibodies. However, its utility in other cohorts may be limited if the prevalence of infection is lower (therefore negatively influencing the positive predictive value), or during acute infection before antibody is detectable.

This study had several limitations. First, the relatively low participation rate raises the possibility that participation bias affected the study results. Specifically, individuals who had already tested positive, or those who had experienced symptoms but not received a positive result, may have been disproportionately motivated to participate in order to have confirmation of infection through antibody testing, leading to overestimation of the attack rate and underestimation of the asymptomatic fraction. Indeed, PCR positivity on 3 April was slightly higher in the study population than among all people on the ship (73% *vs.* 59%), based on another report on this outbreak [23]. According to the same report, only 19% of PCR-positive individuals on board were symptomatic. However, it is unclear whether this number reflects the proportion that was symptomatic only at the time of testing, and if not, for how long cases were followed to determine if they went on to develop symptoms [23]. Furthermore, because symptom data in the present study were recorded about 3 weeks after onset, poor recall may have contributed to overestimation of the asymptomatic fraction, countering some of the effect of the hypothesised participation bias. In addition, some symptoms, including headache or loss of taste or smell were not explicitly queried in our study. Second, the identification of risk factors for symptomatic disease was limited by the small sample size. For example, to detect statistically significant differences, the true relative risk for the effect of sex would need to be at least 3.11 or no more than 0.32, and for the presence of comorbidities at least 2.71 or no more than 0.37. Finally, the study population was mostly aged over 50 years, so caution should be exercised in generalising the findings to the broader population.

To conclude, in this cohort, asymptomatic infection with SARS-CoV-2 was common and the humoral immune response was not dependent on the presence of symptoms. By 3 weeks after disease onset, viral load in respiratory and GI samples was low or undetectable, but serology was useful for confirming prior infection. Demographics and presence of comorbidities were not strong predictors of symptomatic *vs.* asymptomatic disease within this study population. Study of other potential predictors of symptomatic illness, for example genetic or immunologic factors, could inform screening strategies or therapeutics. Research involving longitudinal follow-up of seropositive individuals will help to predict duration of immunity and the utility of sero-surveys in estimating population exposure.

## Data Availability

Readers should contact the authors if they would like to access the data included in this publication.

## Supplementary material

For supplementary material accompanying this paper visit https://doi.org/10.1017/S0950268821000315. click here to view supplementary material
