# Supplementary figures and images for: Symptoms and laboratory manifestations of mild COVID-19 in a repatriated cruise ship cohort
Source: Epidemiol Infect. 2021 Feb 10;149:e44. doi: 10.1017/S0950268821000315 (PMC7900670; doi:10.1017/S0950268821000315)

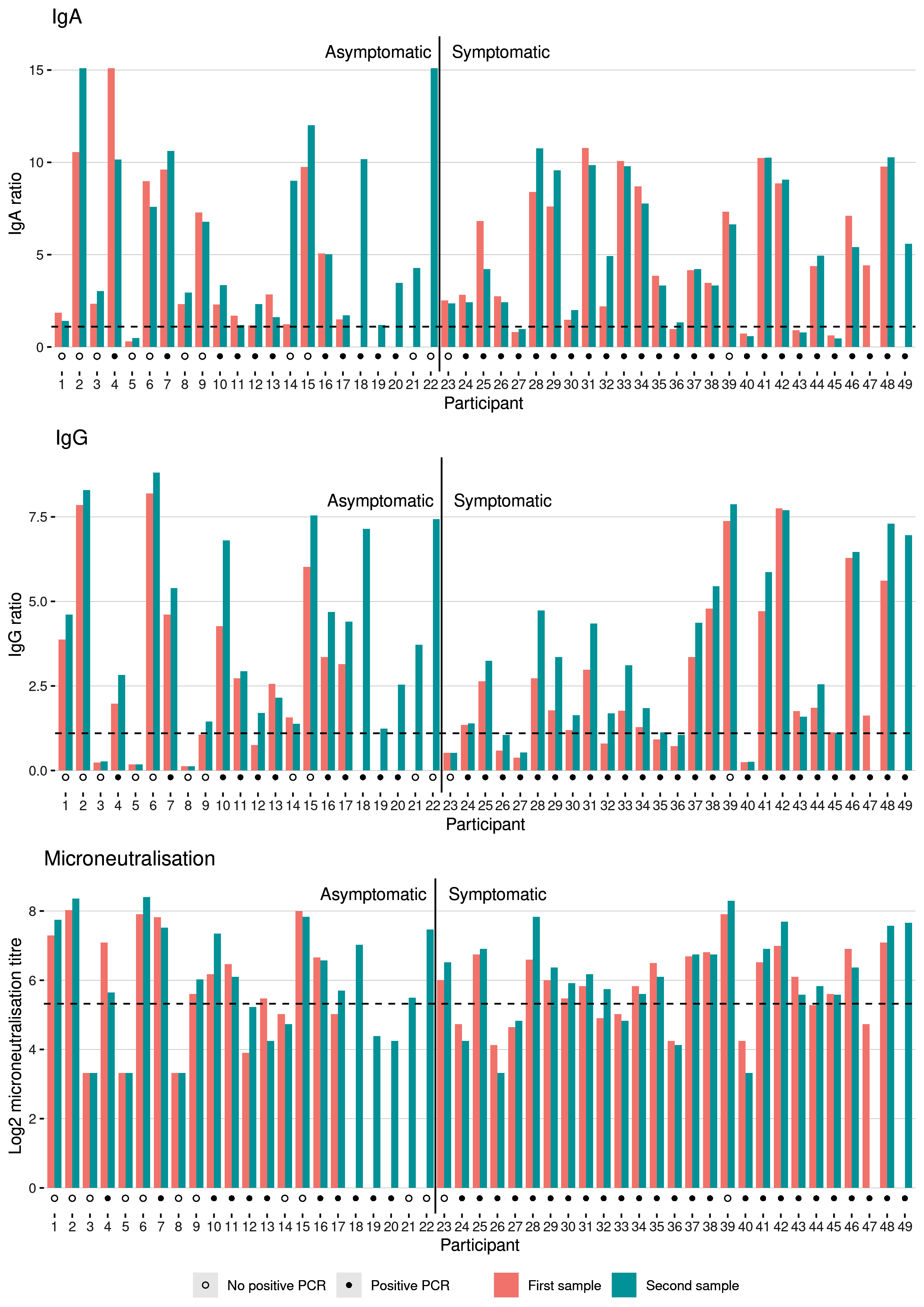

Supplement: Supplementary file 1 [file S0950268821000315sup001.tif]
